# Detection of *Ophiocordyceps sinensis* and Its Common Adulterates Using Species-Specific Primers

**DOI:** 10.3389/fmicb.2017.01179

**Published:** 2017-06-21

**Authors:** Yang Liu, Xiao-yue Wang, Zi-tong Gao, Jian-ping Han, Li Xiang

**Affiliations:** ^1^Identification Center, Institute of Medicinal Plant Development, Chinese Academy of Medical Sciences and Peking Union Medical CollegeBeijing, China; ^2^Artemisinin Research Center, Institute of Chinese Materia Medica, China Academy of Chinese Medical SciencesBeijing, China

**Keywords:** *Ophiocordyceps sinensis*, species-specific primers, identification, mini-barcode, DNA degradation

## Abstract

*Ophiocordyceps sinensis* is a fungus that infects Hepialidae caterpillars, mummifying the larvae and producing characteristic fruiting bodies (stromata) that are processed into one of the most valued traditional Chinese medicines (TCM). The product commands a very high price due to a high demand but a very limited supply. Adulteration with other fungi is a common problem and there is a need to test preparation for the presence of the correct fungus. In the current study, a PCR-based approach for the identification of *O. sinensis* based on a segment of the internal transcribed spacer (ITS) region was developed. The segments is 146-bp in size and is likely to be amplified even in materials where processing led to DNA fragmentation. Primer development was based on the alignment of sequence data generated from a total of 89 samples of *O. sinensis* and potential adulterants as well as sequences date from 41 *Ophiocordyceps* species and 26 Cordyceps species available in GenBank. Tests with primer pair, DCF4/DCR4, demonstrated generation of an amplicon from DNA extracted from *O. sinensis* stromata, but not from extracts derived from adulterants. Species-specific primer pairs were also developed and tested for detection of the common adulterants, *Cordyceps gunnii, Cordyceps cicadae, Cordyceps militaris, Cordyceps liangshanensis* and *Ophiocordyceps nutans*. The collection of primers developed in the present study will be useful for the authentication of preparation claiming to only contain *O. sinensis* and for the detection of fungi used as adulterants in these preparations.

## Introduction

*Ophiocordyceps sinensis* (syn. *Cordyceps sinensis*) is an extremely rare and precious traditional Chinese medicine (TCM) with multiple medicinal values (Wang and Yao, 2011; Quan et al., 2014). This medicinal material is mainly collected in alpine regions over 4,000 m around mountain snowlines on the Tibetan Plateau, the neighboring provinces of the Tibetan autonomous prefectures and the high Himalayas (Yi et al., 2011). As reported in the *New Compilation of Materia Medica, O. sinensis* is beneficial to the kidney. Treatment with *O. sinensis* has also been claimed to have curative effects on several conditions, including erectile dysfunction, bronchial diseases, diabetes, cough and cold, jaundice (Ashok Kumar and Kailash Chandra, 2011; Sirisidthi et al., 2015). The main bioactive components found in *O. sinensis* are adenosine, cordycepin, cordymin, cordysinin, ergosterol, guanosine, myriocin, melanin, lovastatin, and sitosterol (Hui-Chen Lo et al., 2013). Due to strict environmental requirements, *O. sinensis* collected in the field is considered much more pharmacologically valuable than which obtained through culture; however, at present, most of the natural materials is collected by local farmers who do not have the expertise to differentiate between *O. sinensis* and related species. According to one recent study, even some of the *O. sinensis* materials used for study purposes may contain mycelium from other related species (Dong and Yao, 2010). The increasing rate of adulterated *O. sinensis* preparations not only harms consumers and the reputation of Traditional Chinese Medicine (Qin et al., 2011) but also hampers scientific research on this product.

The present identification of *O. sinensis* relies mostly on morphological characteristics, even though this approach has long been controversial. The genus *Ophiocordyceps* was officially defined by Sung et al. (2007) and Chen et al. (2013) and includes *O. sinensis* and similar species distributed within the alpine regions such as *O. gansuënsis, O. crassispora, O. kangdingensis, O. multiaxialis, O. nepalensis*, and others. It is difficult to distinguish these species morphologically (Shrestha et al., 2010) and it is even difficult to differentiate *O. sinensis* from the closely related adulterants, such as *Cordyceps gunnii, Cordyceps cicadae, Cordyceps militaris, Cordyceps liangshanensis* and *Ophiocordyceps nutans*. Chemical methods have also been applied to authenticate *O. sinensis* (Hu et al., 2015; Zhang et al., 2015); however, these method required relative large amounts of sample material. Genetic methods such as analysis of internal transcribed spacer sequences (ITS) and random amplified polymorphic DNA (RAPD)-derived molecular markers have also been used to identify *O. sinensis* (Lam et al., 2015). These methods have focused on detection of *O. sinensis* in untreated fungal material rather than processed materials where DNA degradation or fragmentation can occur (Meissner et al., 2007; Shadi et al., 2011). Therefore, in the present study, a method with specific-species primers was developed in order to increase the probability of detection of *O. sinensis* and common fungal adulterants even in processed samples.

## Materials and methods

### Collection of samples and its sequences

A total of 89 samples of *O*. *sinensis* and its adulterants (*C. gunnii, C. cicadae, C. militaris, C. liangshanensis, O. nutans*) were collected from Qinghai Province, Tibet and Sichuan Province. The details of these samples are listed in Table 1. A total of 131 confirmed ITS sequences of *O. sinensis* were available from previous studies (Chen et al., 2001; Liu et al., 2002; Zhang et al., 2009; Xiang et al., 2014). Additionally, all the known ITS sequences of the genera, *Ophiocordyceps* and *Cordyceps*, were downloaded, and published sequences (sequences in published articles) were selected for further study.

**Table 1:** Information for the samples of *O*. *sinensis* and its counterfeits used in this study.

**Latin name**	**Voucher no**.	**Locality**
Ophiocordyceps sinensis^*^	CSC-1	Qamdo, Tibet China
	CSC-2	Qamdo, Tibet China
	CSC-3	Qamdo, Tibet China
	CSC-4	Qamdo, Tibet China
	CSC-5	Qamdo, Tibet China
	CSC-6	Qamdo, Tibet China
	CSC-7	Qamdo, Tibet China
	CSC-8	Qamdo, Tibet China
	CSC-9	Qamdo, Tibet China
	CSC-10	Qamdo, Tibet China
	CSC-11	Qamdo, Tibet China
	CSC-12	Qamdo, Tibet China
	CSC-13	Qamdo, Tibet China
	CSC-14	Qamdo, Tibet China
	CSC-15	Qamdo, Tibet China
	CSC-16	Qamdo, Tibet China
	CSC-17	Qamdo, Tibet China
	CSC-18	Qamdo, Tibet China
	CSC-19	Qamdo, Tibet China
	CSC-20	Qamdo, Tibet China
	CSC-21	Qamdo, Tibet China
	CSC-22	Qamdo, Tibet China
	CSC-23	Qamdo, Tibet China
	CSC-24	Qamdo, Tibet China
	CSC-25	Qamdo, Tibet China
	CSC-26	Qamdo, Tibet China
	CSC-27	Qamdo, Tibet China
	CSC-28	Qamdo, Tibet China
	CSC-29	Qamdo, Tibet China
	CSC-30	Qamdo, Tibet China
	CSN-1	Yushu, Qinghai China
	CSN-2	Yushu, Qinghai China
	CSN-3	Golog Qinghai China
	CSN-4	Golog Qinghai China
	CSN-5	Golog Qinghai China
	CSN-6	Yushu, Qinghai China
	CSN-7	Yushu, Qinghai China
	CSN-8	Qamdo Tibet China
	CSN-9	Qamdo Tibet China
	CSN-10	Nakchu, Tibet, China
	CSN-11	Nakchu, Tibet, China
	CSN-12	Nakchu, Tibet, China
	CSN-13	Nakchu, Tibet, China
	CSN-14	Nakchu, Tibet, China
	CSN-15	Dege Sichuan China
	CSN-16	Kangting Sichuan China
	CSN-17	Kangting Sichuan China
	CSN-18	Litang Sichuan China
	CSN-19	Litang Sichuan China
	CSN-20	Litang Sichuan China
	CSN-21	Dege Sichuan China
	NQ-1	Nakchu, Tibet China
	NQ-2	Nakchu, Tibet China
	NQ-3	Nakchu, Tibet China
	REG-1	Ruoergai Sichuan China
	REG-2	Ruoergai Sichuan China
	REG-4	Ruoergai Sichuan China
	YS-1	Yushu, Qinghai China
	YS-2	Yushu, Qinghai China
	YS-3	Yushu, Qinghai China
	WZ-1	Unknown, China (market)
	WZ-2	Unknown, China (market)
	WZ-3	Unknown, China (market)
	WZ-4	Unknown, China (market)
	WZ-5	Unknown, China (market)
	WZ-6	Unknown, China (market)
	WZ-7	Unknown, China (market)
	WZ-8	Unknown, China (market)
	WZ-9	Unknown, China (market)
	WZ-10	Unknown, China (market)
	WZ-11	Unknown, China (market)
	WZ-12	Unknown, China (market)
	WZ-13	Unknown, China (market)
	WZ-14	Unknown, China (market)
	WZ-15	Unknown, China (market)
	WZ-16	Unknown, China (market)
	WZ-17	Unknown, China (market)
	WZ-18	Unknown, China (market)
*Ophiocordyceps nutans*	XC-1	Changbai Mountain Nature Reserve Jilin China
	XC-2	Changbai Mountain Nature Reserve Jilin China
*Cordyceps gunnii*	GN-1	Chengtu, Sichuan China (market)
	GN-2	Xizang China (market)
	GN-3	Hubei China (market)
*Cordyceps militaris*	Y-1	Hubei China (market)
*Cordyceps cicadae*	CH-1	Hengduan Mountains Sichuan China
	CH-2	Bozhou Anhui China (market)
	CH-3	Mopan Jiangsu China (market)
*Cordyceps liangshanensis*	LS-1	Sichuan Chian (market)
	LS-2	Sichuan Chian (market)

### DNA extraction, amplification, and sequencing

A total of 20–30 mg stromata of specimens were rinsed with 75% ethanol and milled using a ball-milling machine (Restch, Germany). Genomic DNA was extracted from the resulting powders using a Tiangen Plant DNA Kit (Tiangen Biotech, China). The ITS regions were amplified using an LA Taq polymerase chain reaction (PCR) kit (Takara Biotech Inc.) with the universal primer pairs 5F (5′-GGAAGTAAAAGTCGTAACAAGG-3′)/4R (5′-TCCTCCGCTTATTGATATGC-3′; Li et al., 2013). The PCR mixture contained 0.1 μL of LA Taq (5 U μL^−1^), 2.5 μL of 10 × LA Taq PCR buffer II (Mg^2+^ Plus), 1 μL of dNTP mixture (2.5 mM each), 0.6 μL of each primer (10 μM), and 1 μL (~120 ng) of genomic DNA in a total volume of 25 μL. The samples were amplified using a GeneAmp® PCR system 9700 (Applied Biosystems, Foster City, CA) under the following conditions: initial denaturation at 97°C for 1 min, followed by 30 cycles of denaturation at 97°C for 1 min, annealing at 48°C for 1 min, extension at 72°C for 3 min, and a final elongation step at 72°C for 7 min (Liu et al., 2001).

### Sequence analysis and primer pairs design

The sequences were edited and assembled manually using CodonCode Aligner 5.1.4 (CodonCode Co., USA). Analysis of the ITS sequences database was conducted using CodonCode Aligner software to search species-specific motifs. Potential primers were designed and analyzed using Primer 6.0 software (Glantz, 2005) according to the species-specific motifs. All of the *O. sinensis* ITS sequences were aligned with MEGA (Lewis et al., 2013) software to verify the specificity of the primers for DNA from *O. sinensis* and its adulterants (*C. gunnii, C. cicadae, C. militaris, C. liangshanensis, O. nutans*).

### Preparation of *O. sinensis* decoction and DNA extraction

Each sample was rinsed with 75% ethanol and was then milled using a ball-milling machine (Retsch, Germany); 40 mg of each milled sample was used for the genomic DNA extraction with the Tiangen Plant DNA Kit (Tiangen Biotech, China). Sterilized *O. sinensis* raw materials (stroma) were boiled in 500 mL double-distilled water for 60 and 90 min. The decoction was then dried on a stove by boiling, and 40 mg of the dried decoction was used for DNA extraction with the Tiangen Plant DNA Kit (Tiangen Biotech, China).

### DNA amplification to verify the primer specificity and utility

PCR was performed on DNA extracted from *O. sinensis* decoctions and its adulterants. The reaction was carried out in 25 μL volumes comprised of 2 μL dNTP mixture (2.5 mmol/L), 1.0 μL primers DCF4 /DCR4 (2.5 μmol/L), 4 μL template DNA (~30 ng), 2.5 μL 10 × PCR Buffer (Tiangen Biotech, China), 8 μL Taq DNA polymerase and 6.5 μL sterilized water subject to the following conditions: initial denaturation at 94°C for 3 min, followed by 40 cycles of denaturation at 94°C for 30 s, annealing at 48°C for 30 s, extension at 72°C for 50 s, and a final elongation step at 72°C for 5 min. DNA from boiled materials was also amplified with ITS universal primer pairs to determine if the DNA was still suitable for amplification of larger sequences. PCR with DNA from adulterants was carried out is in 25-μL volumes comprised of 2 μL dNTP mixture (2.5 mmol/L), 2.0 μL primers (2.5 μmol/L), 2 μL template DNA (~30 ng), including pure DNA of adulterants and DNA mixture of *O. sinensis* and each target DNA for the specific primer (at a ratio of 1:1), 2.5 μL 10 × PCR Buffer (Tiangen Biotech, China), 8 μL Taq DNA polymerase and 6.5 μL sterilized water subject to the same conditions.

### Amplification and concentration measurement of diluted DNA

Pure *O. sinensis* DNA was two-fold serially diluted to different multiple to determine the minimum amount of DNA needed for production of amplicons that could be visualized by ethidium bromide staining of agarose gels.

## Results

### Development of unique primers for *O. sinensis*

**Figure 1:**
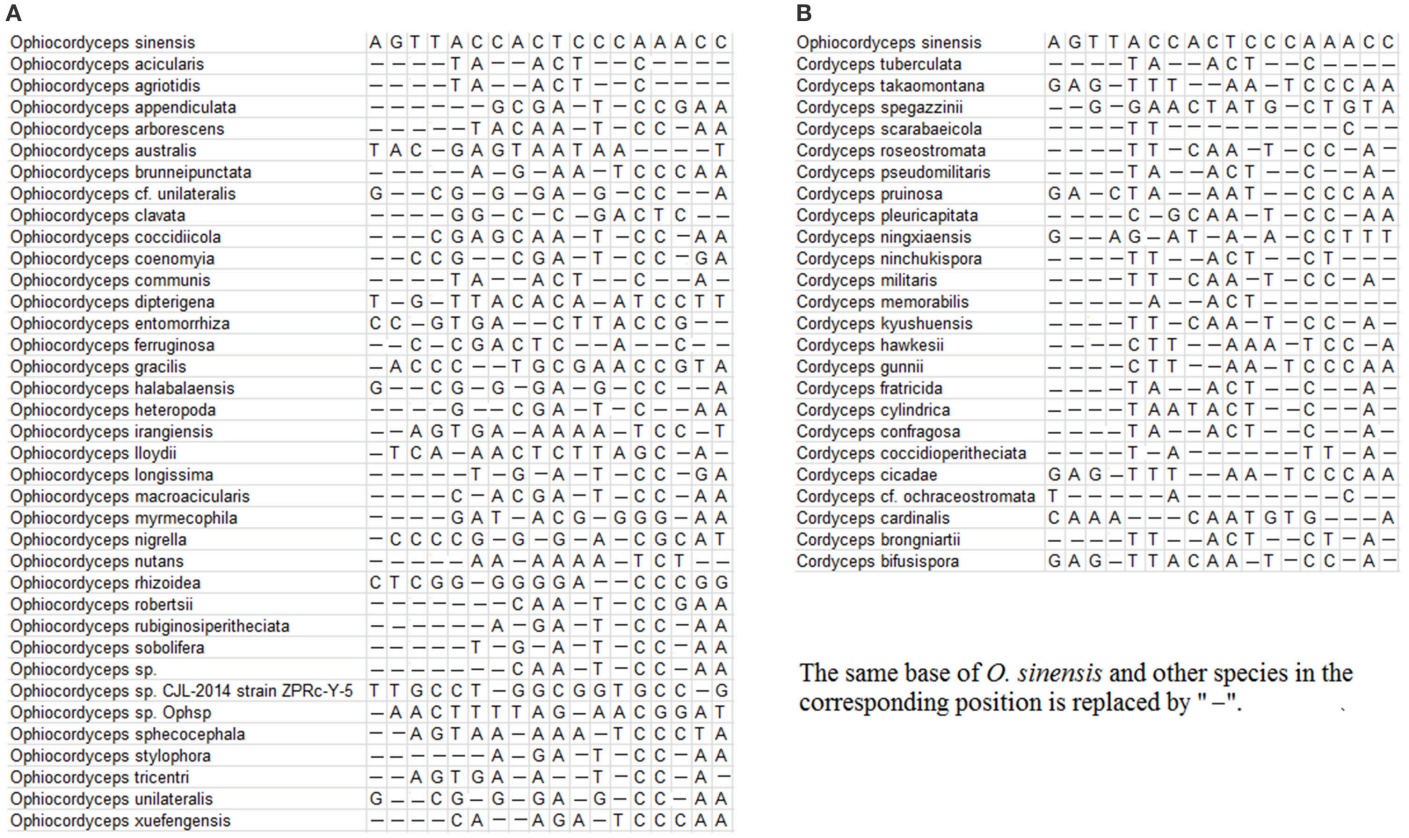
Alignment of DCF4 primer binding regions from congeners and common adulterants. **(A)** DCF4 binding regions from *O. sinensis* and other *Ophiocordyceps* species. **(B)** DCF4 binding regions of *O. sinensis* and other *Cordyceps* species.

A total of 314 identified ITS sequences of *O. sinensis*, including 131 sequences generated in the previous study, were obtained with a length of ~500 bp after alignment; 112 published ITS sequences of 41 different species in *Ophiocordyceps* and 250 published ITS sequences of 26 species in *Cordyceps* were downloaded from the GenBank database. The search for primers specific to the fungal species of interest yielded the primer pairs listed in Table 2. The specificity of the primers DCF4/DCR4 for *O. sinensis* is illustrated in Figures 1A,B. There are at least 3 mismatches between the primers and the corresponding sequences from non- *O. sinensis* species.

**Table 2:** Primers of *O. sinensis* and its counterfeits used for PCR amplification.

**Primer name**	**Species**	**Direction**	**Primer Sequences (5′–3′)**	**Amplicon size**
DCF4	*O. sinensis*	Forward	AGTTACCACTCCCAAACC	146
DCR4	*O. sinensis*	Reverse	TGCTTGCTTCTTGACTGA	146
CCF	*C. cicadae*	Forward	TTACAACTCCCAACCCTTC	209
CCR	*C. cicadae*	Reverse	GATGCCAGAACCAAGAGAT	209
CGF	*C. gunnii*	Forward	TACCTATACTGTTGCTTCGG	203
CGR	*C. gunnii*	Reverse	GATGCCAGAACCAAGAGAT	203
CMF	*C.militaris*	Forward	TGAACATACCTATCGTTGCT	167
CMR	*C.militaris*	Reverse	ATGCCAGAGCCAAGAGAT	167
ONF	*O. nutans*	Forward	AACTCTCCAATTCTCTGTGA	205
ONR	*O. nutans*	Reverse	GCAATTCGCATTACTTATCG	205
CLF	*C. liangshanensis*	Forward	CAGCGGAGGGATCATTAC	219
CLR	*C. liangshanensis*	Reverse	GATGCCAGAACCAAGAGA	219

### Amplification with the species-specific primers DCF4/DCR4 and universal primers

**Figure 2:**
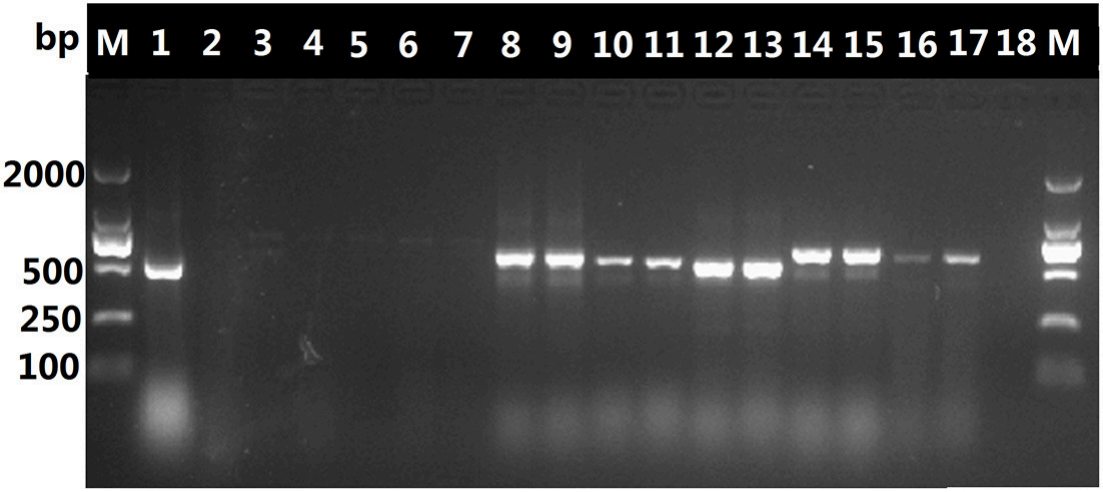
Amplification products generated with ITS universal primer pair 5F/4R and DNA from decoction and adulterants. Lane 1, pure *O. sinensis* DNA (~120 ng); lanes 2, 3, and 4, products from amplification of DNA (~30 ng) isolated from decoction boiled for 60 min; lanes 5, 6, and 7, amplification products from DNA (~30 ng) isolated from decoction boiled for 90 min; results for amplification with pure DNA (~30 ng) from *C. liangshanensis* (lanes 8–9), *C*. *militaris* (lanes 10-11), *O*. *nutans* (lanes 12–13), *C*. *gunnii* (lanes 14–15) and *C*. *cicadae* (lanes 16–17).

**Figure 3:**
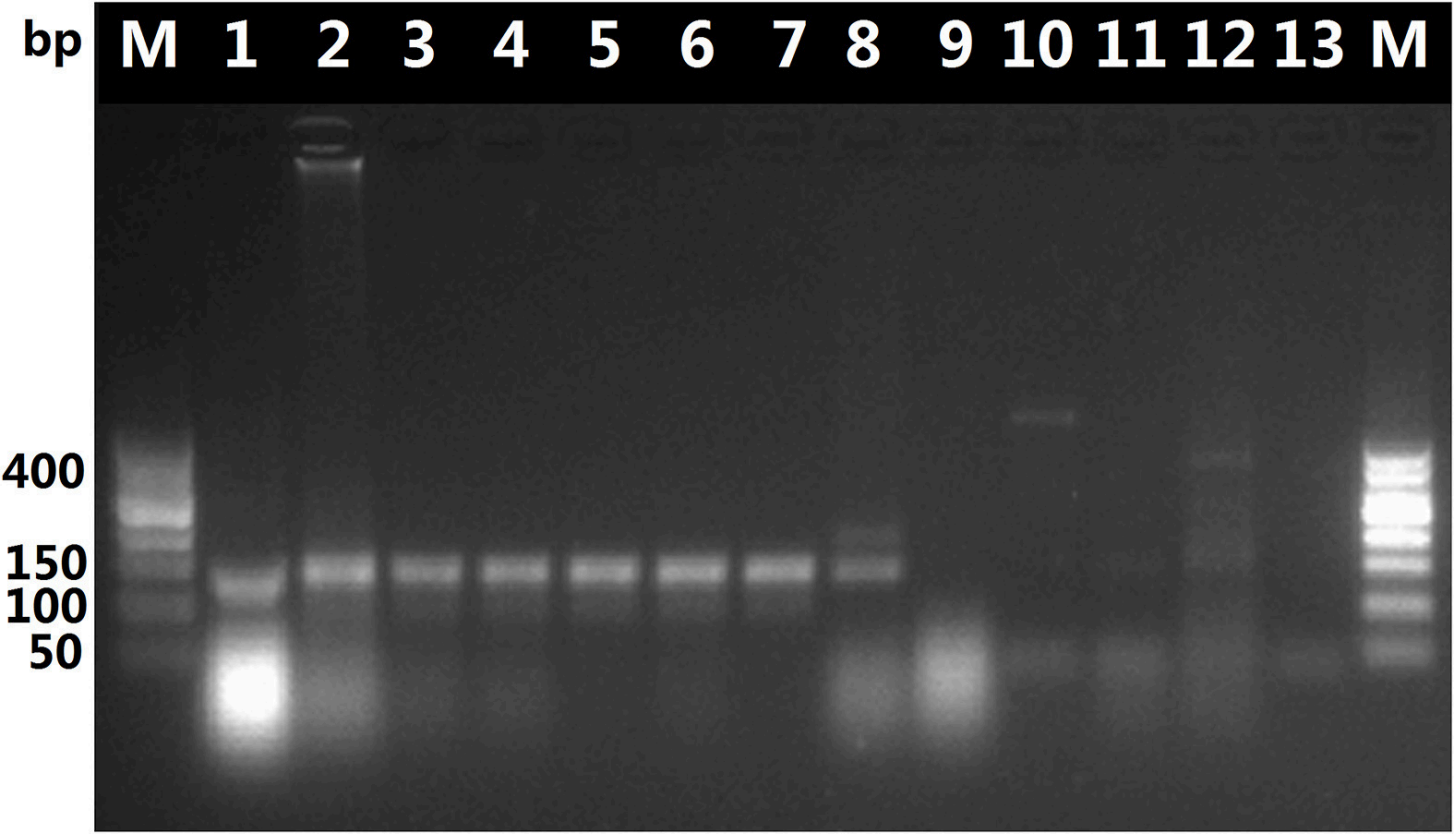
Results of PCR with primers DCF4/DCR4 with DNA isolated from decoction and adulterants. Lane 1, pure *O. sinensis* DNA (~120 ng); lanes 2, 3, and 4, amplification products from DNA (~30 ng) isolated from *O. sinensis* decoction boiled for 60 min; lanes 5, 6, and 7, amplification products from DNA (~30 ng) isolated from decoction boiled for 90 min; lanes 8, 9, 10, 11, and 12, results of PCR with pure DNA (~30 ng) isolated from adulterants *C. liangshanensis, C. militaris, O. nutans, C. gunnii*, and *C. cicadae*, respectively.

The DNA in decoctions boiled for 60 or 90 min was amplified with the universal primer pair 5F/4R, as shown in Figure 2, it appears that the DNA extracted from the *O. sinensis* decoctions was possibly too fractured or otherwise degraded by boiling for 60 or 90 min to serve as template for amplification of the ITS sequence with the universal ITS primers. In contrast, PCR with the *O. sinensis*-specific primer pair yielded DNA that could be visualized after gel electrophoresis. As shown in Figure 3, primers DCF4/DCR4 were also used to amplify five common adulterants (*C. gunnii, C. cicadae, C. militaris, C. liangshanensis, O. nutans*), and no amplification product was seen with DNA obtained from them.

### Amplification with specific primers for five common adulterants

**Figure 4:**
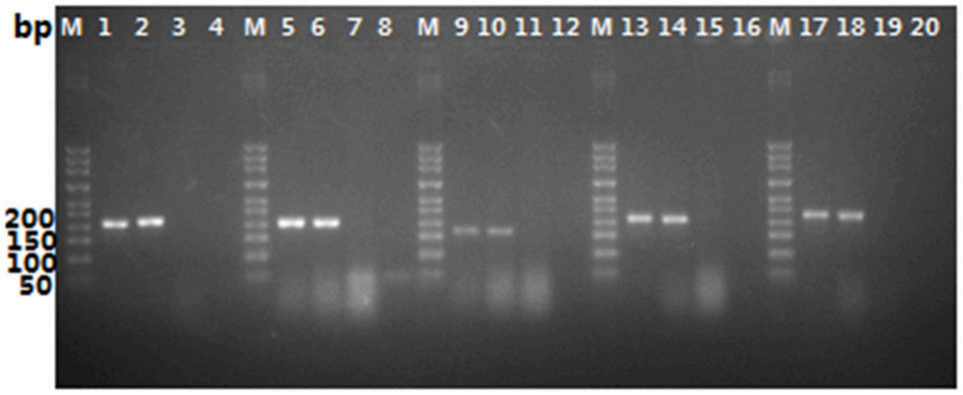
Ampification results for PCR with primer pairs CCF/CCR, CGF/CGR, CMF/CMR, ONF/ONR, and CLF/CLR. CCF/CCR (lane 1–4), CGF/CGR (lane 5–8), CMF/CMR (lane 9–12), ONF/ONR (lane 13–16), CLF/CLR (lane 17–20); lane1, 5, 9, 13, and 17, pure DNA isolated from targeted species (*C. cicadae, C. gunnii, C. militaris, O. nutans*, and *C. liangshanensis*, respectively); lane 2, 6, 10, 14, and 18, mixture of DNA isolated from *O. sinensis* and the targeted species (*C. cicadae, C. gunnii, C. militaris, O. nutans*, and *C. liangshanensis*, respectively) at a ratio of 1:1; lane 3, 7, 11, 15, and 19, pure DNA of *O. sinensis*; lane 4, 8, 12, 16, and 20, negative control.

In order to judge whether the sample were adulterated, five specific primer pairs (CCF/CCR, CGF/CGR, CMF/CMR, ONF/ONR, and CLF/CLR) for common adulterants were designed respectively, according to the conserved motifs obtained by aligning the ITS sequences of the targeted species. As shown in Figure 4, lanes 3, 7, 11, 15, and 19, none of the primers amplified DNA from *O. sinensis*, but they generated PCR products isolated from their respective target organism and in mixtures of *O. sinensis* and target organism DNA. We artificially mixed the DNA of *O. sinensis* and its adulterants, visual PCR products were obtained with each primer pair for each targeted species, as shown in Figure 4. The results showed that the method is suitable for the identification of the mixture of *O. sinensis* and its adulterants.

### Sensitivity of the PCR method

**Figure 5:**
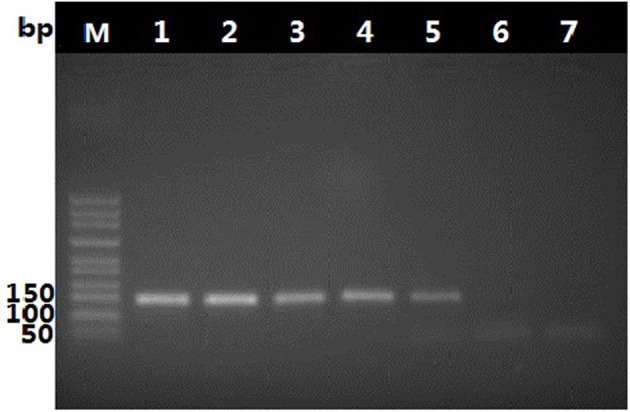
Amplification products generated with primer pair, DCF4/DCR4, and decreasing amount of *O. sinensis* DNA. Lane 1–6: Products generated in a 25-μL reaction mixture with ~120, 60, 30, 15, 8, and 4 ng of *O. sinensis* DNA.

To determine suitable concentration of DNA, the pure *O. sinensis* DNA was two-fold serially diluted to different ratio, from two to six times. Within the scope of dilution times from two to four, the amplification result of diluted DNA showed no obvious difference, as shown in Figure 5, ~8 ng of purified *O. sinensis* DNA in a 25-μL reaction volume were necessary for a band of the PCR products from primers DCF4/DCR4 to be visible in an ethidium bromide-stained gel.

## Discussion

*O. sinensis* has a long history of use as a traditional medicine in China. Due to over exploitation in the past decades, *O. sinensis* has been listed as an endangered species. Naturally produced *O. sinensis* is worth more than gold and because of this high value, adulterants have emerged frequently in recent years, which leads to market instability and a decline in consumer confidence.

DNA-based identification has become important for the identification of medical plants (Ali et al., 2014) as this technique is convenient, generally accurate and usable by people without taxonomic knowledge. The ITS sequence has been recently selected as the official marker for fugal genetic identification by the Consortium for the Barcode of Life (Das and Deb, 2015). ITS sequences amplification was used to identify fungi from soils or water as an environmental DNA barcode (Bellemain et al., 2010). Dentinger et al. compared the suitability of cytochrome oxidase subunit I (CO1) gene and ITS sequences for mushrooms and fern allies identification and determined that ITS-based identification is superior (Dentinger et al., 2011). Our previous study focused on the identification of raw *O. sinensis* materials based on ITS sequences (Xiang et al., 2014); however, the suitability for use with processed TCMs was not determined. The current study demonstrated that it is possible to apply PCR-based methodology to determine the presence of *O. sinensis* DNA in TCMs. Therefore, in another previous study, we proposed a mini-barcoding technique using short barcodes with a relatively high identification specificity for TCM (Liu et al., 2016), demonstrating immediate relevance to both science, industry and consumers. Further studies on mini-barcoding for the identification of TCM are necessary and beneficial. Although ITS sequences are commonly used to identify fungi, the requirement for relatively intact DNA to obtain complete ITS amplicons can make this approach difficult when DNA is extracted from processed samples whose DNA might have been degraded. We hypothesized that amplification of shorter regions of ITS might be possible with DNA from processed samples since Lo et al. were able to amplify a 88-bp fragment from TCM material after it had been boiled for 120 min (Lo et al., 2015). The present study also showed that a 146-bp fragment could be amplified from DNA extracted from processed *O. sinensis* samples, whereas amplification of the entire ITS region was not possible. For the first time, a specific primer pair is proposed and is proved to be a very efficient tool for the identification of *O. sinensis* and its adulterants.

The specificity of this primer pair, allows authentication of *O. sinensis* materials by PCR and amplicon detection along without the need for sequencing. Therefore, analysis times and costs are reduced. The assays can potentially be further simplified and expedited by utilizing isothermal recombinase polymerase amplification and an amplicon detection method that does not involve gel electrophoresis (Del Río et al., 2014). The application of the *O. sinensis*-specific primer pair along with the five primer pairs targeting DNA from common adulterants should allow determining if a sample said to only contain *O. sinensis* actually also, or exclusively, contains adulterants added inadvertently or deliberately.

## Conclusion

In this study, a species-specific primer pair that amplifies a 146-bp sequence unique to the ITS region of *O. sinensis* was established. Besides that, five specific primer pairs for common adulterated species were also established. The method developed in this study provides users with an easy authentication method and may make a major contribution to the detection of counterfeit products of *O. sinensis* in the markets. In conclusion, this method can greatly expand the molecular identification of DNA-degraded materials and result in the rapid authentication of *O. sinensis* and its common adulterants among all its congeners with high accuracy, specificity and low cost.

## Author contributions

JH designed this study. JH and LX provided experimental data. YL analyzed the raw data and drafted the manuscript. All authors helped to finish the manuscript and approved the final manuscript.

### Conflict of interest statement

The authors declare that the research was conducted in the absence of any commercial or financial relationships that could be construed as a potential conflict of interest.

## References

[B1] Ali M. A. Gyulai G. Hidvégi N. Kerti B. Al Hemaid F. M. Pandey A. K. (2014). The changing epitome of species identification - DNA barcoding. Saudi J. Biol. Sci. 21, 204–231. 10.1016/j.sjbs.2014.03.003 24955007 PMC4061418

[B2] Ashok Kumar P. Kailash Chandra S. (2011). Traditional uses and medicinal potential of *Cordyceps sinensis* of Sikkim. J. Ayurveda Integr. Med. 2, 9–13. 10.4103/0975-9476.78183 21731381 PMC3121254

[B3] Bellemain E. Carlsen T. Brochmann C. Coissac E. Taberlet P. Kauserud H. (2010). ITS as an environmental DNA barcode for fungi: an *in silico* approach reveals potential PCR biases. BMC Microbiol. 10:189. 10.1186/1471-2180-10-189 20618939 PMC2909996

[B4] Chen Y. Q. Ning W. Qu L. H. Li T. H. Zhang W. M. (2001). Determination of the anamorph of *Cordyceps sinensis* inferred from the analysis of the ribosomal DNA internal transcribed spacers and 5.8S rDNA. Biochem. Syst. Ecol. 29, 597–607. 10.1016/S0305-1978(00)00100-9 11336809

[B5] Chen Z. H. Dai Y. D. Yu H. Yang K. Yang Z. L. Yuan F.. (2013). Systematic analyses of *Ophiocordyceps lanpingensis* sp. nov., a new species of Ophiocordyceps in China. Microbiol. Res. 168, 525–532. 10.1016/j.micres.2013.02.010 23578962

[B6] Das S. Deb B. (2015). DNA barcoding of fungi using Ribosomal ITS Marker for genetic diversity analysis: a review. Int. J. Pure Appl. Biosci. 3, 160–167. Available online at: http://www.ijpab.com/form/2015%20Volume%203,%20issue%203/IJPAB-2015-3-3-160-167.pdf

[B7] Del Río J. S. Yehia A. N. Acero-Sánchez J. L. Henry O. Y. O'Sullivan C. K. (2014). Electrochemical detection of *Francisella tularensis* genomic DNA using solid-phase recombinase polymerase amplification. Biosens. Bioelectron. 54, 674–678. 10.1016/j.bios.2013.11.035 24334283

[B8] Dentinger B. T. Didukh M. Y. Moncalvo J. M. (2011). Comparing COI, and ITS as DNA barcode markers for mushrooms and allies (Agaricomycotina). PLoS ONE 6:e25081. 10.1371/journal.pone.0025081 21966418 PMC3178597

[B9] Dong C. H. Yao Y. J. (2010). On the reliability of fungal materials used in studies on *Ophiocordyceps sinensis*. J. Ind. Microbiol. 38, 1027–1035. 10.1007/s10295-010-0877-4 20922458

[B10] Glantz S. A. (2005). Primer of Biostatistics: Statistical Software Program Version 6.0. New York, NY: McGraw-Hill Medical.

[B11] Hu H. Xiao L. Zheng B. Wei X. Ellis A. Liu Y. M. (2015). Identification of chemical markers in *Cordyceps sinensis* by HPLC-MS/MS. Anal. Bioanal. Chem. 407, 8059–8066. 10.1007/s00216-015-8978-6 26302964 PMC4596796

[B12] Hui-Chen Lo C. H. Lin F. -Y. Hsu T. -H. (2013). A systematic review of the mysterious caterpillar fungus *Ophiocordyceps sinensis* in Dong-ChongXiaCao (冬蟲夏草 Dōng Chóng Xià Cǎo) and related bioactive ingredients. J. Trad. Complement. Med. 3, 16–32. 10.1016/S2225-4110(16)30164-X PMC3924981 24716152

[B13] Lam K. Y. Chan G. K. Xin G. Z. Xu H. Ku C. F. Chen J. P.. (2015). Authentication of *Cordyceps sinensis* by DNA analyses: comparison of ITS sequence analysis and RAPD-derived molecular markers. Molecules 20, 22454–22462. 10.3390/molecules201219861 26694332 PMC6332357

[B14] Lewis P. O. Kumar S. Tamura K. Nei M. Lewis P. O. (2013). MEGA6: molecular evolutionary genetics analysis version 6.0. Mol. Biol. Evol. 30, 2725–2729. 10.1093/molbev/mst197 24132122 PMC3840312

[B15] Li X. Song J. Xin T. Zhu Y. Shi L. Xu X.. (2013). DNA barcoding the commercial Chinese caterpillar fungus. FEMS Microbiol. Lett. 347, 156–162. 10.1111/1574-6968.12233 23927075

[B16] Liu Y. Wang L. Wang X. Chen X. Han J. Pang X. (2016). A nucleotide signature for the identification of american ginseng and its products. Front. Plant Sci. 7:319. 10.3389/fpls.2016.00319 27047504 PMC4796032

[B17] Liu Z. Y. Liang Z. Q. Liu A. Y. Yao Y. J. Hyde K. D. Yu Z. N. (2002). Molecular evidence for teleomorph-anamorph connections in Cordyceps based on ITS-5.8S rDNA sequences. Mycol. Res. 106, 1100–1108. 10.1017/S0953756202006378

[B18] Liu Z. Y. Yao Y. J. Liang Z. Q. Liu A. Y. Pegler D. N. Chase M. W. (2001). Molecular evidence for the anamorph—teleomorph connection in *Cordyceps sinensis*. Mycol. Res. 105, 827–832. 10.1017/S095375620100377X

[B19] Lo Y. T. Ming L. Shaw P. C. (2015). Identification of constituent herbs in ginseng decoctions by DNA markers. Chin. Med. 10, 1–8. 10.1186/s13020-015-0029-x 25657815 PMC4318153

[B20] Meissner C. Bruse P. Mueller E. Oehmichen M. (2007). A new sensitive short pentaplex (ShoP) PCR for typing of degraded DNA. Forensic Sci. Int. 166, 121–127. 10.1016/j.forsciint.2006.04.014 16814503

[B21] Qin S. Zhao J. Liu X. Hua C. Shu Y. Yue G. (2011). Current market state investigation and strategy of *Cordyceps sinensin* (Berk.) Sacc. Lishizhen Med. Mater. Med. Res. 22, 1236–1237.

[B22] Quan Q. M. Wang Q. X. Zhou X. L. Li S. Yang X. L. Zhu Y. G.. (2014). Comparative phylogenetic relationships and genetic structure of the caterpillar fungus *Ophiocordyceps sinensis* and its host insects inferred from multiple gene sequences. J. Microbiol. 52, 99–105. 10.1007/s12275-014-3391-y 24500473

[B23] Shadi S. Xin Z. Janzen D. H. Winnie H. Jean- François L. Jacobus L. M. (2011). Pyrosequencing for mini-barcoding of fresh and old museum specimens. PLoS ONE 6:e21252 10.1371/journal.pone.0021252 21818256 PMC3144868

[B24] Shrestha B. Zhang W. Zhang Y. Liu X. (2010). What is the Chinese caterpillar fungus *Ophiocordyceps sinensis* (Ophiocordycipitaceae)? Mycol. Int. J. Fungal Biol. 1, 228–236. 10.1080/21501203.2010.536791

[B25] Sirisidthi K. Kosai P. Jiraungkoorskul W. (2015). Antihyperglycemic activity of *Ophiocordyceps sinensis*: a review. Indian J. Agric. Res. 49, 400–406. 10.18805/ijare.v49i5.5801

[B26] Sung G.-H. Hywel-Jones N. L. Sung J.-M. Luangsa-ard J. J. Shrestha B. Spatafora J. W. (2007). Phylogenetic classification of *Cordyceps* and the clavicipitaceous fungi. Stud. Mycol. 57, 5–59. 10.3114/sim.2007.57.01 18490993 PMC2104736

[B27] Wang X. L. Yao Y. J. (2011). Host insect species of *Ophiocordyceps sinensis*: a review. Zookeys 127, 43–59. 10.3897/zookeys.127.802 PMC3175130 21998547

[B28] Xiang L. Son J. Xin T. Zhu Y. Shi L. Xu X. (2014). DNA barcoding the commercial chinese Caterpillar Fungus, in The 14th National Conference on Traditional Chinese Medicine and Nature Medicine Paper Abstract (Beijing), 156–162.

[B29] Yi L. Wang X. L. Lei J. Yi J. Hui L. Jiang S. P. (2011). A survey of the geographic distribution of *Ophiocordyceps sinensis*. J. Microbiol. 49, 913–919. 10.1007/s12275-011-1193-z 22203553

[B30] Zhang J. Wang P. Wei X. Li L. Cheng H. Wu Y.. (2015). A metabolomics approach for authentication of *Ophiocordyceps sinensis* by liquid chromatography coupled with quadrupole time-of-flight mass spectrometry. Food Res. Int. 76, 489–497. 10.1016/j.foodres.2015.07.025 28455029

[B31] Zhang Y. Xu L. Shu Z. Liu X. An Z. Mu W.. (2009). Genetic diversity of *Ophiocordyceps sinensis*, a medicinal fungus endemic to the Tibetan Plateau: Implications for its evolution and conservation. BMC Evol. Biol. 9:290. 10.1186/1471-2148-9-290 20003548 PMC2805636

